# Transcriptome analysis revealed the regulation of gibberellin and the establishment of photosynthetic system promote rapid seed germination and early growth of seedling in pearl millet

**DOI:** 10.1186/s13068-021-01946-6

**Published:** 2021-04-11

**Authors:** Bingchao Wu, Min Sun, Huan Zhang, Dan Yang, Chuang Lin, Imran Khan, Xiaoshan Wang, Xinquan Zhang, Gang Nie, Guangyan Feng, Yanhong Yan, Zhou Li, Yan Peng, Linkai Huang

**Affiliations:** College of Grassland Science and Technology, Sichuan Agricultural University, Chengdu, 6111130 China

**Keywords:** Pearl millet, Seed germination, Seedling growth, Transcriptome, Hormone signal transduction

## Abstract

**Background:**

Seed germination is the most important stage for the formation of a new plant. This process starts when the dry seed begins to absorb water and ends when the radicle protrudes. The germination rate of seed from different species varies. The rapid germination of seed from species that grow on marginal land allows seedlings to compete with surrounding species, which is also the guarantee of normal plant development and high yield. Pearl millet is an important cereal crop that is used worldwide, and it can also be used to extract bioethanol. Previous germination experiments have shown that pearl millet has a fast seed germination rate, but the molecular mechanisms behind pearl millet are unclear. Therefore, this study explored the expression patterns of genes involved in pearl millet growth from the germination of dry seed to the early growth stages.

**Results:**

Through the germination test and the measurement of the seedling radicle length, we found that pearl millet seed germinated after 24 h of swelling of the dry seed. Using transcriptome sequencing, we characterized the gene expression patterns of dry seed, water imbibed seed, germ and radicle, and found more differentially expressed genes (DEGs) in radicle than germ. Further analysis showed that different genome clusters function specifically at different tissues and time periods. Weighted gene co-expression network analysis (WGCNA) and Kyoto Encyclopedia of Genes and Genomes (KEGG) enrichment analysis showed that many genes that positively regulate plant growth and development are highly enriched and expressed, especially the gibberellin signaling pathway, which can promote seed germination. We speculated that the activation of these key genes promotes the germination of pearl millet seed and the growth of seedlings. To verify this, we measured the content of gibberellin and found that the gibberellin content after seed imbibition rose sharply and remained at a high level.

**Conclusions:**

In this study, we identified the key genes that participated in the regulation of seed germination and seedling growth. The activation of key genes in these pathways may contribute to the rapid germination and growth of seed and seedlings in pearl millet. These results provided new insight into accelerating the germination rate and seedling growth of species with slow germination.

**Supplementary Information:**

The online version contains supplementary material available at 10.1186/s13068-021-01946-6.

## Background

Seed germination is a complicated physiological process that is affected by both the internal development of the seed and changes in the external environment [1]. Germination begins with the absorption of water by seed and ends with the initiation of hypocotyl elongation [1]. After seed germination, growth begins with the appearance of the radicle and continues with seedling growth [2]. In order to avoid competition for land with crops, bioenergy crops are usually planted on marginal land where resources are very limited. Plants in this environment tend to compete for sunlight, air, and water. Due to the fragility of seedling in early growth stages, their swiftness and robustness will impact later development and yield [3]. Therefore, the rapid germination of seed is very important because it can improve the ability of plants to compete with other species in the field. Moreover, the rapid germination of seed involves many physiological and biochemical reactions, which are often controlled by genes. It is necessary to understand how rapid seed germination occurs in order to improve the scale of plant cultivation and economic benefits, as well as provide a reference accelerating germination of species with slow seed germination.

Fossil fuels have been used for a long time to fulfill the demand for energy and their use still dominates the market; however, the negative effects of fossil fuels cannot be ignored. Fossil fuels pose an obvious risk to environmental governance and energy security; therefore, a major portion of the world’s population has shifted their attention from fossil fuels to bioenergy [4]. Pearl millet (*Pennisetum glaucum* (L.) R. Br.), an important cereal/bioenergy crop contributes to a variety of application worldwide. Pearl millet is widely planted and has fast growth, high yield, and strong resistance to various environmental stressors. Pearl millet contains easy-to-extract fermented sugars, with up to 243 kg ha^−1^ of fermentable sugar output [5], that can be used to produce bioethanol; thus, many researchers regard it to have the potential for bioenergy production [6–8]. In addition, pearl millet is one of the parents of the important energy plant known as hybridized *Pennisetum*, and it is the sixth most predominant cereal crop in the world [9]. Through our germination experiments, we found that the seed germination and seedling growth rate of pearl millet is extremely fast, significantly faster than maize (*Zea mays* L.), rice (*Oryza sativa* L.), orchardgrass (*Dactylis glomerata* L.), and switchgrass (*Panicum virgatum* L.; Additional file 1: Figure S1 and Additional file 2: Table S1). Although pearl millet is considered a crop with fast seed germination, the reason behind its fast germination rate is still unknown.

Plant hormones, including gibberellic acid (GA), auxin, and cytokinins (CTKs), are organic signaling molecules that are produced by via metabolic pathways in plants and they actively participate in various physiological processes. Plant hormones can function at the place of synthesis and can also be transported to distant parts of the plant [10]. Plant hormones can act independently or in coordination with each other to regulate the growth and development of plants [11]. Many studies have found that abnormalities in their synthesis pathways or signal transduction pathways will cause defects in plant growth and development [12]. Among these hormones, abscisic acid (ABA) and GA are the dominant factors related to seed dormancy and germination, respectively [13]. ABA promotes seed dormancy, while GA helps the seed to break dormancy, promoting germination [14, 15]. A previous study has reported that the lower GA content in the seed of mutant *ga1*, which had blocked GA synthesis, had incomplete germination even under normal conditions [16]. Another study revealed that fast-growing switchgrass seedlings accumulated higher GA content than slow-growing seedlings [17]. Auxin mainly regulates the development of plant leaves and roots [18, 19]. Some studies have found that auxin can maintain root stem cells by inducing the accumulation of transcription factor PLT1/2 [20]. In addition, auxin can induce the growth of adventitious roots in rice [21]. CTKs are involved in the development of plant roots. The overexpression of CTK A response factors can promote the growth of *Arabidopsis* roots [22], which has also been observed in rice [23]. Some evidence has shown that the growth of adventitious roots in rice significantly decreases after treatment with inhibitors of CTK synthesis, indicating that CTKs behave like auxin and are involved in regulating the development of adventitious roots [24]. However, whether these hormones regulate the seed germination and seedling growth of pearl millet through signal transduction pathways is still unknown.

The growth of plants is inseparable from photosynthesis, and the factors that affect photosynthesis may also influence the growth and development of plants [25]. Previously, it has been described that increasing the daily light integral (DLI) within a certain range can improve the growth of plants, promote the accumulation of nutrients, and accelerate the developmental process [26, 27]. As an important part of the photosynthetic system, photosynthetic antenna proteins contain different pigments and have varied absorption characteristics for different light, thus increasing the efficiency of photosynthesis [28]. The early growth rate of pearl millet seedlings is very rapid, so we can speculate that this is closely related to the sufficient energy supply of pearl millet seedlings. However, it remains unknown whether or not the photosynthetic system has been formed and activated at this stage.

Transcriptome sequencing technology has become an effective means to understand the relationship between gene expression patterns and phenotypes [29–32]. Transcriptome sequencing at different time points, tissues of germinated seed, and seedling growth in pearl millet will help us to identify the key genes involved at this stage. For example, a study on the transcriptome dynamics of leaves during the germination of corn seed found that gene clusters involved in hormonal metabolism and signal transduction have different expressions at different time points [33]. In this study, we conducted a seed germination experiment and found that pearl millet seed germinated at ≤ 24 h after imbibition, and the radicle elongation was faster than that of the germ. Based on the transcriptome data, there were more DEGs in the radicle than in the germ. A WGCNA was performed on all DEGs, which revealed that different gene clusters were time and tissue specific. Finally, we found that the high expression of key genes involved in hormone signal transduction, photosynthesis, photosynthesis-antenna proteins, and the brassinosteroid synthesis pathway may be related to the rapid germination and growth of seed and seedling in pearl millet. The sharp increase of GA content after seed imbibition confirmed the activation of the gibberellin signal transduction pathway and resulted in rapid seed germination. These results provide a reference for accelerating the seed germination of species with slow seed germination and seedling growth.

## Results

### The germination rate and seedling growth is extremely fast in pearl millet

**Fig. 1:**
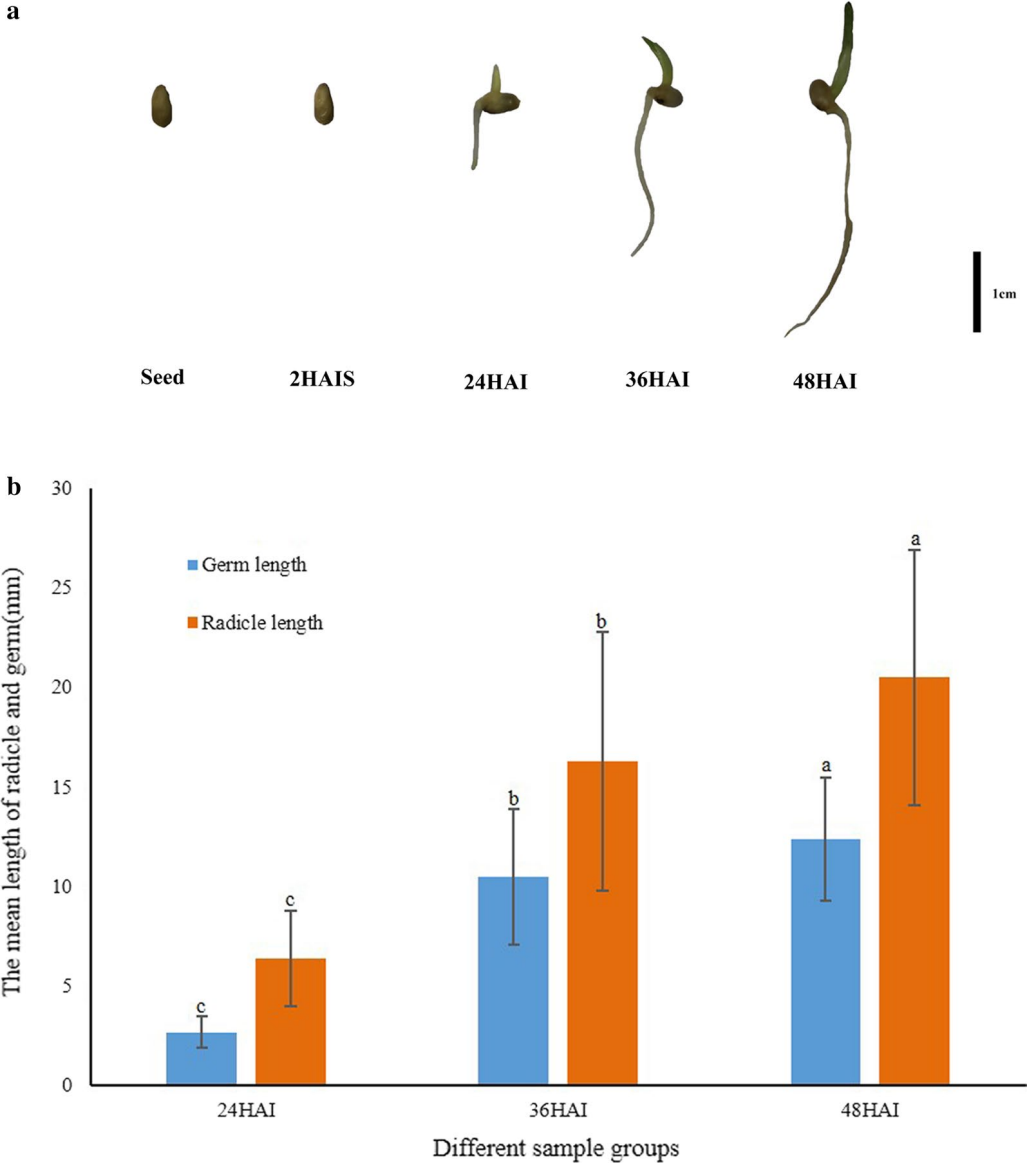
Changes of pearl millet seed after imbibition. **a** Morphological changes of pearl millet seed from dry seed to seedling. **b** The lengths of germ and radicle at different time points. Different lowercase letters indicate significant difference at *P* < 0.05

In our studies on seed germination, we found that pearl millet seedling grew very rapidly under suitable temperature and light conditions (Fig. 1, Additional file 1: Figure S1 and Additional file 2: Table S1). We speculated that a series of rapid physiological and biochemical reactions occurred during the very early stage of seedling development. In order to determine the reasons for the rapid germination rate, we focused on five time points in the early germination period: (1) the dry seed, (2) 2 h after imbibition (HAI; still in seed form), (3) 24 HAI (radicle and germ appeared), (4) 36 HAI, and 48 HAI. We measured the length of the germ and radicle of seedling at 24, 36, and 48 HAI to characterize the growth rate of seedling.

The results showed that the germination rate in pearl millet seed is very rapid (Fig. 1, Additional file 2: Table S2). At ≤ 24 h after seed imbibition, the length of the germ and radicle reached the germination standard, and growth rate of the radicle was faster than that of the germ, especially during the period from 24 to 36 h (Fig. 1a). From 24 to 48 h, the seedlings experienced very rapid growth and there were significant differences in the germ length and radicle length between time points (Fig. 1b). These results suggested that the pearl millet seed gained some advantages after imbibition, allowing the seed to rapidly germinate and grow. This indicates that we can study the transcriptome differences during this period, which will provide insight into the mechanism of pearl millet seed germination and seedling growth.

### More DEGs in the radicle than the germ

Through transcriptome sequencing, a total of 810,182,656 raw reads were generated from 24 samples, ranging from 34,402,297 to 42,543,864 reads. The original data have been uploaded to the NCBI database under project number PRJNA670183. After filtering the original data, we obtained 779,909,975 clean data, ranging from 33,395,607 to 41,346,841 clean reads. The GC content of all samples ranged from 52.57 to 57.59%, the Q20 ranged from 96.93 to 98.04%, and the Q30 ranged from 92.01 to 94.66% (Additional file 2: Table S3), indicating a high sequencing quality and that the obtained data can be used for subsequent analyses.

**Fig. 2:**
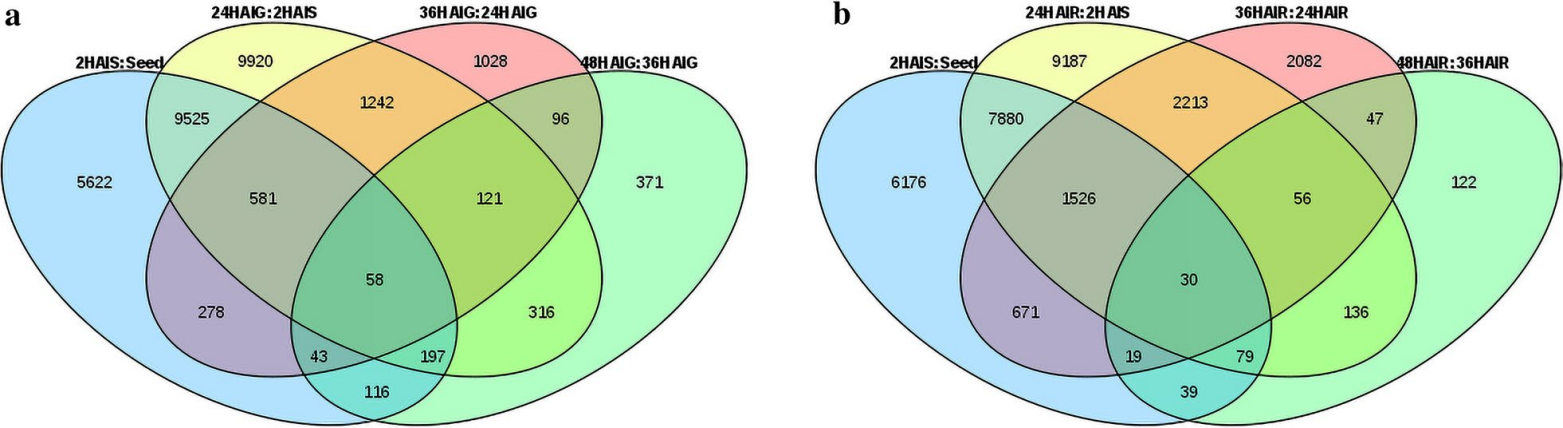
Overlap of differentially expressed genes in different comparison groups: **a** germ, **b** radicle

We used the full-length transcriptome sequence of pearl millet as a reference sequence for alignment and the calculation of gene expression [8]. Great consistency between different biological replicates of each sample is a prerequisite to ensure the reliability of subsequent bioinformatics analysis results. Therefore, we calculated Pearson's correlation coefficient for different samples and found that the three biological replicates between each sample were strongly correlated, and the correlation coefficients were all greater than 0.9 (*P* value < 0.01) (Additional file 1: Figure S2, Additional file 2: Table S4). In order to understand the dynamics of gene expression changes in the seed, germ, and radicle at different time points, we divided the 16 comparison groups into four categories for DEGs (Table 1). A total of 29,514 DEGs were identified in the germ, of which only 58 genes were shared among all time points, whereas a total of 30,263 DEGs were identified in the radicle, of which only 30 genes were shared among all time points (Fig. 2). It is worth noting that in both the radicle and the germ, in the 24 HAI and 2 HAI comparison groups (24 HAIG:2 HAIS and 24 HAIR:2 HAIS), there were more down-regulated genes than up-regulated genes and the total number of DEGs was the largest, whereas all other comparison groups had more up-regulated genes than down-regulated genes. This may indicate that gene expression is very strong at 2 HAIS. We also found that the number of DEGs in the radicle was greater than that in the germ in other comparison groups, except for in the 24 HAI:2 HAI and the 48 HAI:36HAI comparison group after seed imbibition. This indicated that the gene expression in the radicle was more active than the germ, which may explain why the radicle appeared earlier than the germ and the faster growth rate. Because the elongation of the radicle was significantly greater than that of the germ at 36 HAI compared with 24 HAI, we investigated the DEGs of the germ and the radicle in the 36 HAI:24 HAI groups, and found that there were 6644 DEGs in the radicle, which was higher than 3447 DEGs in the germ. Then, we compared the germ and radicle at three time points and found that the number of DEGs between the radicle and germ was 12,697 in the 36 HAIG:36 HAIR group, which was significantly higher than the 7239 and 8546 of 24 HAI and 48 HAI. This may explain why the elongation of the radicle is much greater than that of the germ at 36 HAI.

**Table 1:** Summary of DEGs among 16 comparison groups

Sort	Comparison groups	Number of up-regulated genes	Number of down-regulated genes	Total number of DEGs
Seed	2HAIS:Seed	10,595	5825	16,420
Germ	24HAIG:Seed	8807	6468	15,275
	36HAIG:Seed	10,026	7082	17,108
	48HAIG:Seed	11,049	5879	16,928
	24HAIG:2HAIS	10,043	11,917	21,960
	36HAIG:24HAIG	2457	990	3447
	48HAIG:36HAIG	805	513	1318
Radicle	24HAIR:Seed	9828	7324	17,152
	36HAIR:Seed	12,646	6458	19,104
	48HAIR:Seed	12,853	6806	19,659
	24HAIR:2HAIS	9703	11,404	21,107
	36HAIR:24HAIR	4526	2118	6644
	48HAIR:36HAIR	386	142	528
Germ:radicle	24HAIG:24HAIR	3699	3540	7239
	36HAIG:36HAIR	5538	7159	12,697
	48HAIG:48HAIR	4938	3608	8546

### The regulation mechanism of seed germination is specific at different stages

**Fig. 3:**
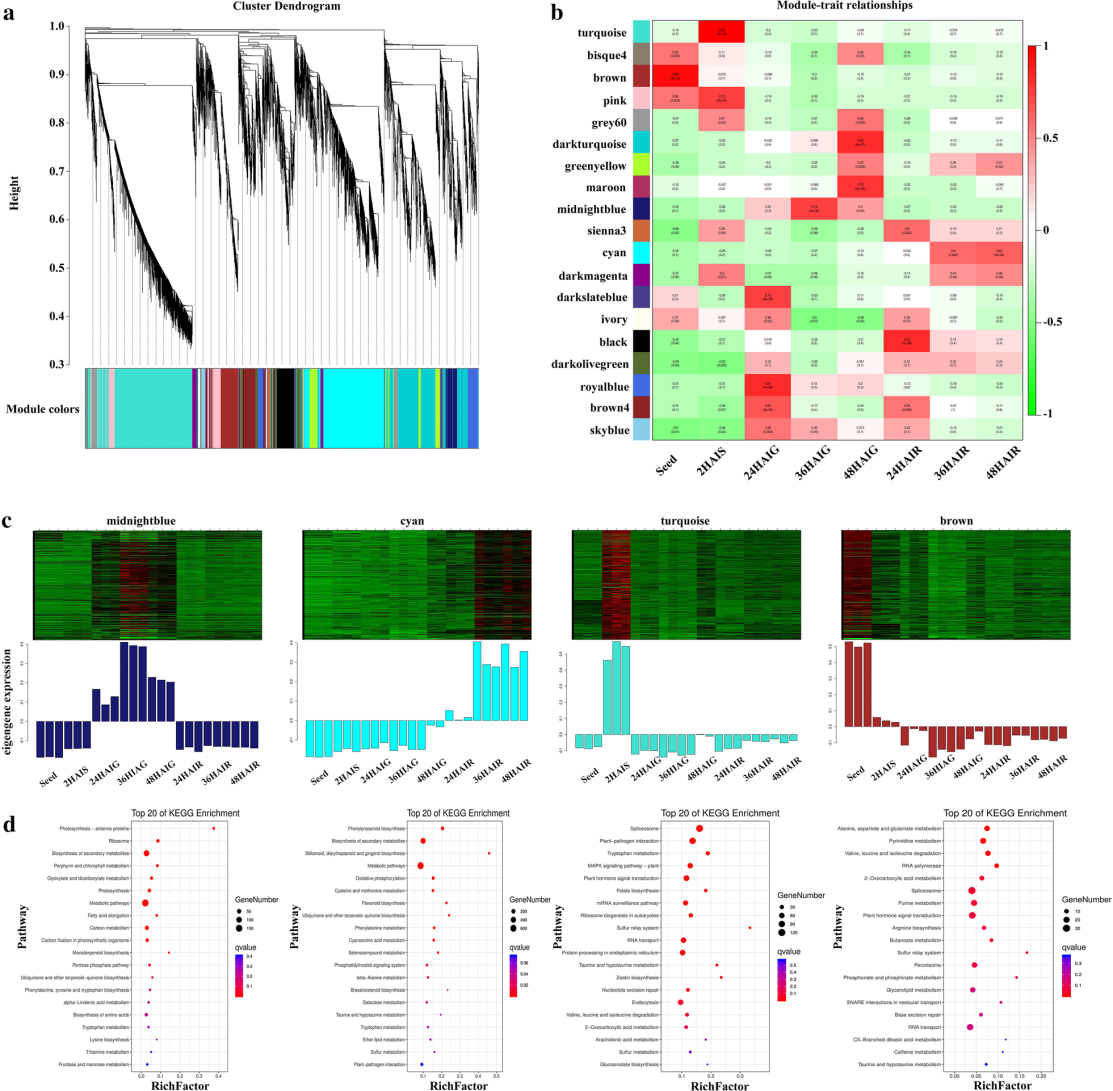
Weighted gene co-expression network analysis (WGCNA) results of DEGs. **a** Cluster dendrogram. **b** Module–trait relationships. **c** Expression heat map and expression level of genes in the module. **d** KEGG enrichment of genes in the module

To better identify the DEGs related to different time points and their expression trends in the radicle and germ, the standardized expression data of 24,307 genes were analyzed using a gene co-expression network analysis from 24 samples (three biological replicates). A total of 19 modules, which is a cluster of highly related genes, were generated, with each color representing a module (Fig. 3a, b). We found four modules with specific time or tissue expression: “midnightblue”, “cyan”, “turquoise”, and “brown” (Fig. 3c). The brown module contains 1409 genes that are mainly expressed in dry seed; the turquoise module contains 5577 genes that are mainly expressed at 2 HAIS. The midnight blue module contains 902 genes that are mainly expressed in germ at 24, 36, and 48 HAI, and the cyan module contains 4498 genes that are mainly expressed in the radicle at 36 and 48 HAI.

In order to further understand the biological functions of these four modules, we performed a KEGG analysis on the genes in these four modules (Fig. 3d). The genes in the brown module are significantly enriched in alanine, aspartate, and glutamate metabolism (ko00250); pyrimidine metabolism (ko00240); valine, leucine, and isoleucine degradation (ko00280); and RNA polymerase (ko03020). The genes in the turquois module are significantly enriched in 13 pathways including plant hormone signal transduction (ko04075) and MAPK signaling pathway-plant (ko04016). The genes in the midnight blue module are enriched in 11 pathways such as photosynthesis-antenna proteins (ko00196); porphyrin and chlorophyll metabolism (ko00860); photosynthesis (ko00195); carbon metabolism (ko01200); and carbon fixation in photosynthetic organisms (ko00710). The genes in the cyan module are significantly enriched in 17 pathways including phenylpropanoid biosynthesis (ko00940), flavonoid biosynthesis (ko00941), and brassinosteroid biosynthesis (ko00905) (Fig. 3d, Additional file 2: Table S4). It is worth noting that there are few pathways shared by genes in the four modules (Additional file 1: Figure S3, Additional file 2: Table S5). The turquoise and cyan modules shared two pathways: taurine and hypotaurine metabolism (ko00430) and tryptophan metabolism (ko00380). Similarly, the midnight blue and cyan modules shared two pathways: biosynthesis of secondary metabolites (ko01110) and metabolic pathways (ko01100). The brown module did not share pathways with any module. The above results indicated that the function of genes in the module had strong tissue and time specificity, and different genes function at different stages, indicating that exploring the regulation mechanism of each stage can help us better understand the reasons for rapid germination in pearl millet seed.

### Functional analysis of candidate genes related to seed germination and seedling growth

#### The signal transduction of GA, auxin, and cytokinin promotes the rapid germination of pearl millet seed

**Fig. 4:**
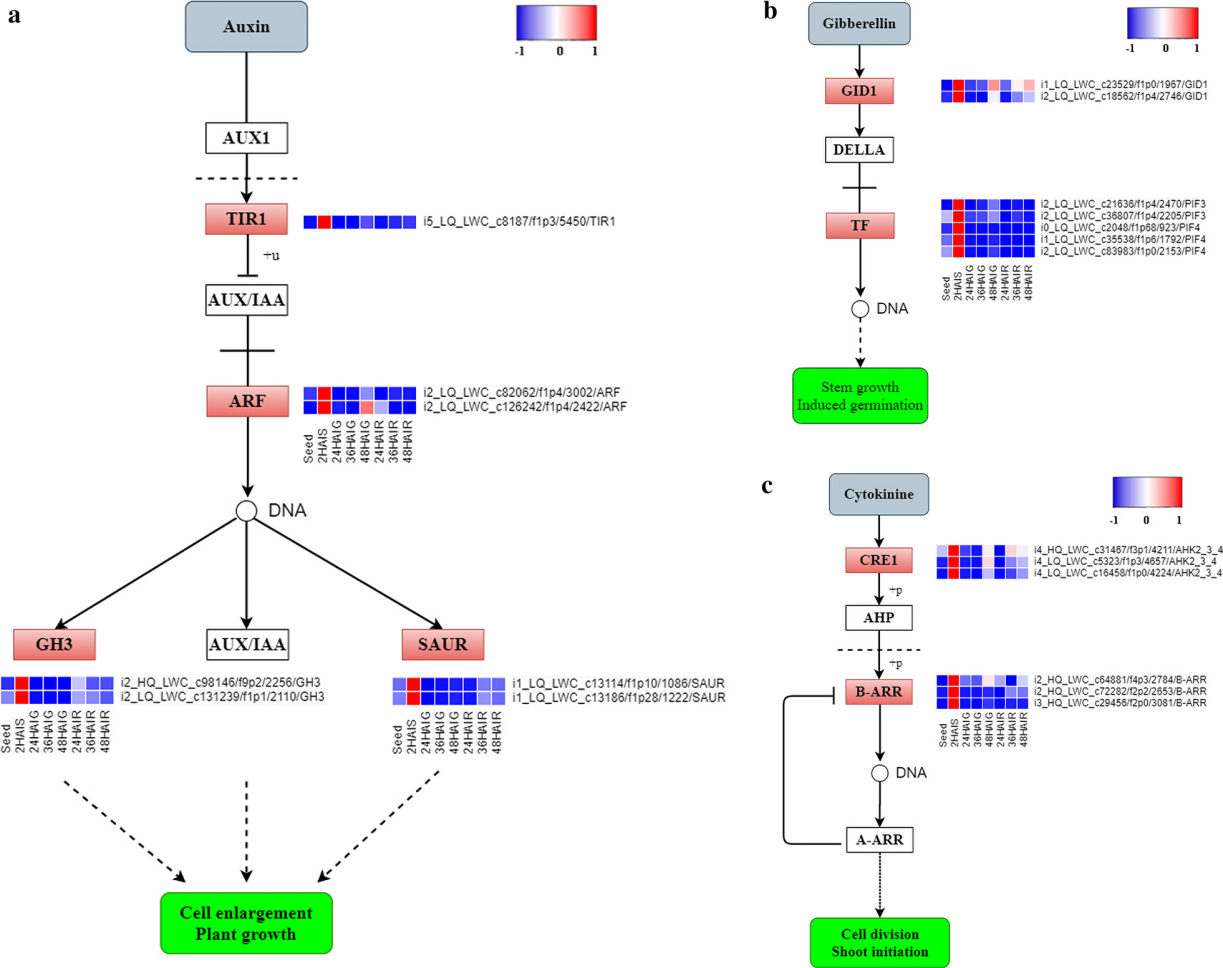
The expression pattern of key genes in hormone signal transduction pathway. **a** The pathway in auxin signal transduction. **b** The pathway in gibberellin signal transduction. **c** The pathway in cytokinin signal transduction. The red rectangle indicates that the gene is enriched in the pathway. The expression data are the TPM values of the samples, red color indicates upregulated expression, and blue indicates downregulated expression

**Fig. 5:**
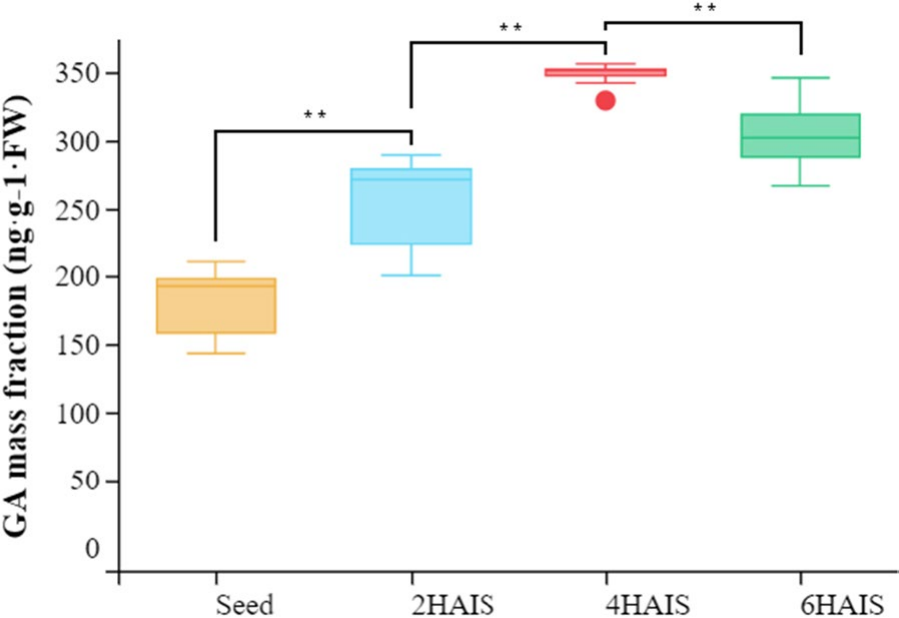
Gibberellin content at different time points. ** Indicates significant difference at *P* < 0.01

GA is a type of diterpene compound that plays an important role in the growth and development of plants, and numerous studies have shown that it regulates the process of seed germination. When GA works through the signal transduction pathway, the receptor GID1 first senses gibberellin, and then combines with the DELLA protein to form a GID1/GA/DELLA complex, which relieves the inhibition of DELLA on key downstream regulatory factors such as PIF3 and PIF4, and then regulates various biological processes [34, 35]. Our results found that the expression of two *GID1* genes (i1_LQ_LWC_c23529/f1p0/1967 and i2_LQ_LWC_c18562/f1p4/2746) reached the highest level 2 HAI, and the same expression trend was also observed in the two *PIF3* genes (i2_LQ_LWC_c21636/f1p4 /2470 and i2_LQ_LWC_c36807/f1p4/2205) and three *PIF4* genes (i0_LQ_LWC_c2048/f1p68/923, i1_LQ_LWC_c35538/f1p6/1792 and i2_LQ_LWC_c83983/f1p0/2153) (Fig. 4b). Considering that GA is the main hormone related to seed germination, and the signal transduction pathway of this hormone is highly active, we determined the GA content in the early stage after seed imbibition. We found that the content of GA in dry seed was the lowest, began to increase after imbibition, and reached the highest level at 4 HAIS. The difference between every two time points was extremely significant (*P* < 0.01) (Fig. 5, Additional file 2: Table S6), indicating that a large amount of GA was synthesized immediately after seed imbibition and the downstream signal transduction pathway was also stimulated to promote rapid seed germination.

Auxin was the earliest discovered plant hormone. It is a general term for a class of compounds that include indole acetic acid (IAA) and have similar physiological effects as indole acetic acid. Auxin is involved in many biological and physiological processes including growth and development of roots and leaves [18, 20, 36, 37]. In the current study, we found that the *TIR1* gene (i5_LQ_LWC_c8187/f1p3/5450) and two *ARFs* genes (i2_LQ_LWC_c82062/f1p4/3002 and i2_LQ_LWC_c126242/f1p4/2422) were highly expressed at 2 HAIS. In addition, some studies have shown that the downstream gene, *GH3*, of this pathway was up-regulated by auxin, but it involved the degradation of auxin, so it resulted in negative feedback on auxin [38]. It is generally believed that GH3 plays an important role in auxin homeostasis [38]. SAUR is thought to be involved in auxin-regulated cell expansion, and it has also been found to be highly expressed in hypocotyl elongation [39, 40]. We found that two *GH3* genes (i2_HQ_LWC_c98146/f9p2/2256 and i2_LQ_LWC_c131239/f1p1/2110) and two *SAUR* genes (i1_LQ_LWC_c13114/f1p10/1086 and i1_LQ_LWC_c13186/f1p28/1222) have similar expression patterns (Fig. 4a).

CTKs are involved in the regulation of plant cell division, growth, and development of tissues and organs. The signal transduction pathway of CTKs is first sensed by the histidine receptor kinase CRE1 (AHK2_3_4) to autophosphorylate histidine, and then the phosphate group is transferred to the aspartic acid residue in the self-receptive region. Then the phosphate group on the aspartic acid residue of the receptor is transferred to the histidine residue of the cytoplasmic histidine phosphorylation transfer protein (AHP). Finally, the phosphorylated histidine transfer protein enters the nucleus and transfers phosphate groups to A or B response factors (ARRs), among which B response factors (ARR-B) have transcription factor activity and can initiate downstream gene expression after phosphorylation [41–43]. Our research found that the expression levels of three *CRE1* genes (i4_HQ_LWC_c31467/f3p1/4211, i4_LQ_LWC_c5323/f1p3/4657 and i4_LQ_LWC_c16458/f1p0/4224) and three *ARR-B* genes (i2_HQ_LWC_c64881/f4p3/2784, i2_HQ_LWC_c72282/f2p2/2653 and i3_HQ_LWC_c29456/f2p0/3081) peaked at 2 HAIS (Fig. 4c).

We found that in the signal transduction pathways of GA, auxin, and CTKs, the expression level of genes reached the highest level at 2 HAIS in pearl millet. Therefore, we speculated that after the seed absorb water for a short time, the hormone signal transduction in the seed becomes active, which promoted the germination of pearl millet seed.

#### The formation of the photosynthetic system promotes the rapid growth of pearl millet seedlings

**Fig. 6:**
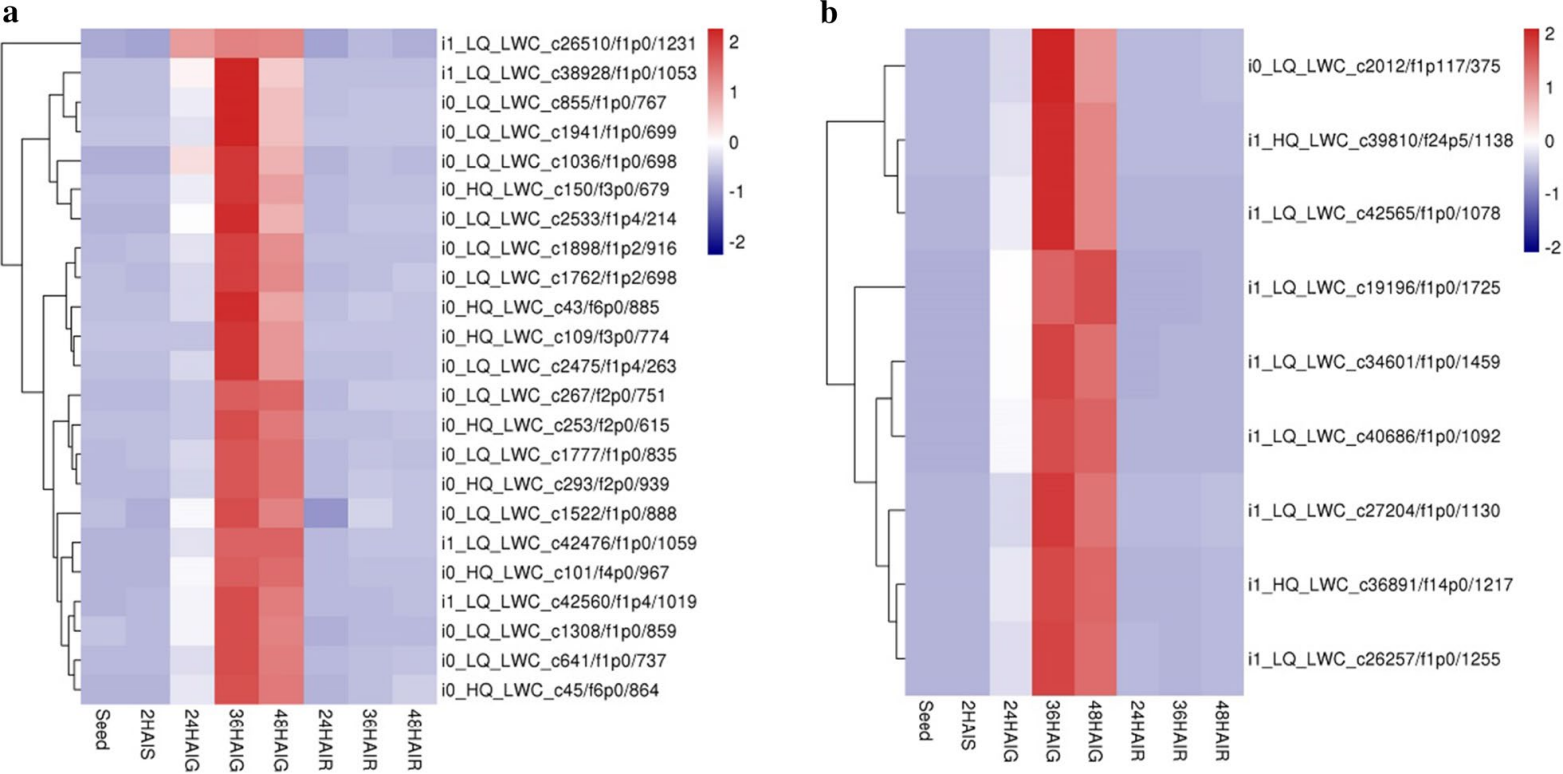
Heat map of genes expression related to light pathway. **a** The genes in the photosynthetic pathway. **b** The genes in the photosynthesis-antenna proteins pathway. The expression data are the TPM values of the samples, red color indicates upregulated expression, and blue indicates downregulated expression

Photosynthesis plays a very important role in the growth and development of plants. It includes two light reactions: Photosystem I and Photosystem II. Photosystem I mostly produce negative oxidation–reduction reactions in nature, and to a large extent determines the amount of global enthalpy in the living system. Photosystem II produces an oxidant with a redox potential that is sufficient to oxidize H_2_O, which is a very abundant substrate that can ensure an almost unlimited source of electrons for life on Earth [44]. Both systems are multi-subunit supramolecular complexes, including a core complex and a peripheral antenna system [44, 45]. In plants, the peripheral antennas are all made up of the light-harvesting complex (LHC). LHCIs (Lhca) and the PSI core form a PSI–LHCI complex, and LHCIIs (Lhcb) and the PSII core form a PSII–LHCII complex. The antenna system has a different pigment composition; therefore, it has different light absorption characteristics [28]. The light-harvesting complex (LHCII) in PSII is the most abundant membrane protein on Earth. It participates in the first step of photosynthesis, absorbs and transmits solar energy for photosynthesis on the chloroplast membrane, and plays a role in regulating photosynthesis and photoprotection [46, 47]. Our results found that in the photosynthetic pathway, a total of 23 genes had the highest expression levels at 36 HAIG (Fig. 6a). In the photosynthesis-antenna protein pathway, in addition to LHCA5, the other four light-harvesting complexes in LHCI (*LHCA1*, i0_LQ_LWC_c2012/f1p117/375; *LHCA2*, i1_LQ_LWC_c27204/f1p0/1130; *LHCA3*, i1_HQ_LWC_c39810/f24p5/1138; *LHCA4*, i1_LQ_LWC_c34601/f1p0/1459) were significantly enriched and had the highest expression at 36 HAI in the germ. *LHCB1* (i1_LQ_LWC_c19196/f1p0/1725, i1_LQ_LWC_c40686/f1p0/1092, and i1_LQ_LWC_c42565/f1p0/1078), *LHCB4* (i1_HQ_LWC_c36891/f14p0/1217), and LHCB5 (i1_LQ_LWC_c26257/f1p0/1255) in LHCII also showed the same expression pattern (Fig. 6b). These results showed that the genes involved in photosynthesis and antenna protein genes in the germ were highly expressed at 36 HAI. The increased expression levels allowed the plant to generate a lot of energy for growth and utilization, and the antenna protein system promoted the transmission of solar energy and improved the effectiveness of photosynthesis. Therefore, we speculated that the rapid formation of the photosynthetic system helped the pearl millet to more effectively produce energy for growth and development, which is an important reason for the rapid growth of the seedlings in pearl millet.

#### Brassinosteroids promote radicle elongation

Brassinosteroids (BRs) are widely distributed plant hormones that play an important role in almost all of the growth processes of plants, including regulating the elongation and division of cells [48]. In rice that lacks BRs, its growth and development is affected, resulting in stunted growth [49]. The BR-insensitive mutants of *Arabidopsis thaliana* showed many defects during growth and development, including short plants and reduced apical dominance [48]. The interaction of BRs and auxin mediated by BRX (BREVIS RADIX) in *Arabidopsis* roots is necessary for optimal root growth. The phenotype of the *brx* mutant is caused by the lack of root-specific BRs. This defect affects approximately 15% of the root gene expression levels of all *Arabidopsis* genes, but the expression levels of these genes can be restored by BR treatment [50], suggesting that the normal level of BRs is essential for the development of plants, especially roots.

We found that the key enzymes in the biosynthetic pathway of BRs include *CPD* (i2_HQ_LWC_c41220/f2p0/2056 and i2_LQ_LWC_c11071/f1p0/2085), *DET* (i3_LQ_LWC_c34585/f1p1/4026), and *CYP92A6* (i1_LQ_LWC_c8195/f1p3/1783 and i1_LQ_LWC_c36100/f1p0/1831), which were all enriched and expressed in the radicle (Additional file 1: Figure S4). In addition, *D2* (i1_LQ_LWC_c2765/f2p1/2032) and *CYP734A1* (i2_LQ_LWC_c108886/f1p0/2200), which inactivate BRs through hydroxylation to maintain the steady state of BRs, also had higher expression levels in the radicle (Additional file 1: Figure S4). The genes that synthesize BRs and maintain BR homeostasis in the radicle were very active, which ensured normal level of BRs. We speculated that this may explain why the elongation of the radicle was significantly higher than that of the germ.

## Discussion

Our results showed that individual pearl millet seed reached the germination standard at slightly different times under the same environmental conditions. However, nearly all seeds reached the germination standard 24 HAI. Therefore, pearl millet can be regarded as a species that is representative of plants with rapid seed germination. Translating these findings to the field, the rapid germination of pearl millet may allow it to not be easily eroded or encroached upon by weeds. In order to explore the growth rate of pearl millet seedlings, we measured the length of germ and radicle at 24, 36, and 48 HAI. The result showed that there were extremely significant differences between each time point, and the average germ and radicle lengths reached 12.4 mm and 20.5 mm at 48 HAI, respectively (Additional file 2: Table S2). Switchgrass, another plant that can be used as a biofuel, reaches 20–30 mm tall in seedlings after 10 days of growth [11]. The growth rate of pearl millet seedlings is much faster than that of switchgrass. Therefore, pearl millet can also be regarded as representative of rapid seedling growth.

In previous studies, many researchers have reported that GA can promote seed germination [51–53]. A previous study found that the LOL1/bZIP58 complex in rice can promote the synthesis of gibberellins and enhance the germination rate and speed of rice seed [54]. The *Arabidopsis* GA deletion mutant *ga1* cannot germinate without the application of exogenous GA [16, 55]. In the signal transduction pathway of GA, the DELLA protein is the main suppressor, which binds to key downstream regulatory factors to inhibit the signal transduction of GA [34]. A previous study found that DELLA protein enhanced the degradation of PIF3, inhibiting the elongation of the hypocotyl of *Arabidopsis* [35]. In addition to promoting seed germination, GA can also promote root growth by degrading DELLA protein [56]. In this study, we found that the GA signal transduction pathway was very active at 2 HAI, especially the genes *GID1*, *PIF3*, and *PIF4*, which were highly expressed, while the inhibitory DELLA protein gene was not expressed. In addition, we measured the GA content of pearl millet seed in the early stage after imbibition and found that a large amount of GA was accumulated. In fact, the GA content accumulated by pearl millet was much higher than that in switchgrass [17]. Considering the slow germination of switchgrass seed, we speculate that the large amount of GA was accumulated in the early stage after seed imbibition and is one of the reasons for the rapid germination of pearl millet seed.

In addition, to promote leaf and root development, auxin plays an active role in plant growth and development, such as regulating tissue differentiation, organogenesis, morphogenesis, apical dominance, and flowering period [57–60]. A recent study has shown that auxin stimulates the abscisic acid signals to induce seed dormancy [61]. Therefore, the balance of auxin content is essential for seed germination and seedling development. In our study, we found that *GH3*, which promotes the degradation of auxin, was highly expressed in the auxin signal transduction pathway. The expression of this gene was induced by auxin and had negative feedback regulation. We speculated that this gene promotes the maintenance of a balanced level of auxin, which neither promotes seed dormancy nor hinders other aspects of plant growth and development.

CTKs play a major role in plant growth including promoting cell division, inducing bud differentiation, removing apical advantages, and enhancing plant resistance. Many studies have also found that CTKs efficiently promotes seed development, increases the seed setting rate, and breaks seed dormancy [62]. The earliest discovered and purified CTKs was zeatin, which was isolated from immature corn seed [63]. In the process of seed germination, GA cannot reach the point of action due to cell compartment barriers, but CTKs can change the permeability of the membrane to promote GA to function [62]. In this study, we found that after 2 HAI, the CTKs signal transduction pathway was very active, and the receptor kinase CRE1 and class B response factor ARR-B genes were highly expressed. In addition, the biosynthetic pathway of zeatin was also significantly enriched at this stage (Additional file 2: Table S5). Zeatin is the main active ingredient of CTKs and its biosynthesis allows for CTKs to function. Therefore, we speculate that the activation of the zeatin biosynthesis pathway and the signal transduction pathway of CTKs may promote the rapid germination of pearl millet seed.

Previous studies have reported that during the germination of tobacco and *Arabidopsis* seed, the release of dormancy involves the light/gibberellin pathway and results in testa rupture [10, 64, 65]. In our study, in addition to the abovementioned GA-related pathways, light-related pathways have also been detected. Ferredoxin, which acts as a regulator, can improve the efficiency of the dark reaction [66]. We determined the expression level of gene *petF* (i0_HQ_LWC_c109/f3p0/774, i0_LQ_LWC_c1036/f1p0/698, and i1_LQ_LWC_c26510/f1p0/1231), which encodes ferredoxin and found that it was very active in germ, showing a peak at 36 HAI. At the same time, the photosystem subunits are highly expressed (Fig. 6), which all play an important role in photosynthesis. For example, the PSI-H subunit (i0_LQ_LWC_c641/f1p0/737) is necessary for the transition of the energy state in the light system, which is a dynamic mechanism for plants to quickly respond to changes in light [67]. The PSI-D subunit in *Arabidopsis* is encoded by the *PsaD* gene (i0_HQ_LWC_c150/f3p0/679 and i0_LQ_LWC_c1898/f1p2/916). The *PsaD* mutant is seedling-lethal in *Arabidopsis*, illustrating that *PsaD* plays an important role in maintaining the stability of Photosystem I [68]. In addition, studies have shown that Photosystem II oxygen-promoting protein 2 (*PsbP*, i1_LQ_LWC_c42560/f1p4/1019 and i1_LQ_LWC_c38928/f1p0/1053) and Photosystem II oxygen-promoting protein 3 (*PsbQ*, i0_LQ_LWC_c1522/f1p0/888) have specific and important roles in coordinating the activity of the donor and acceptor sides of PSII and stabilizing the active form of the PSII-light-harvesting complex II (LHCII) supercomplex [69]. We speculate that the formation of the photosynthetic system at the early seedling stage provides sufficient energy for seedling development, which accelerates the growth of seedlings in pearl millet.

## Conclusion

The germination experiments conducted in this study showed that the germination and seedling growth rate of pearl millet are rapid. Under suitable temperature and light conditions, seed can germinate at 24 HAI. In addition, seedlings experienced rapid growth during 24–36 HAI. Therefore, we regard pearl millet as a representative plant for rapid seed germination and seedling growth. The WGCNA analysis showed that the functions of different gene clusters were highly organized and time specific, indicating that different genes were involved in the regulation of seed germination, as well as the growth of the germ and radicle. The KEGG enrichment analysis showed that differential expression of key genes in the GA, auxin, and CTK signal transduction pathways, photosynthesis, photosynthesis-antenna proteins, and brassinosteroids biosynthesis pathway regulated the germination and growth of pearl millet seedlings. This study provided a reference for accelerating seed germination and the growth and development of seedlings. Our findings can be used as a transcriptome data resource for comparison and analysis in future studies.

## Methods

### Plant materials

The seed of pearl millet cv. “Tifleaf 3” were used in our study and provided by key Laboratory Department of Grassland Science, Sichuan Agricultural University (Wenjiang, Sichuan, China). For the experiment, 200 seeds with uniform size and shape, and without damage were randomly selected. First, 30 seeds were randomly selected as the dry seed group (seed), and the remaining 170 seeds were soaked in distilled water and shaken at 200 rpm for 10 min to fully imbibe. Then 170 seeds were putted in a petri dish with a double-layer filter paper as a germination bed and all of them were maintained in an incubator at 30 °C/25 °C (day/night) with a photoperiod of 16 h/8 h (day/night). After 2 h, 30 seeds were randomly selected as the 2 h after seed imbibition group (2HAIS). Further, seedlings have been formed after 24, 36 and 48 h seed imbibition. Thirty individual plants were randomly selected to measure the length of germ and radicle, respectively. After the measurement, the germ and radicle were separated as the 24, 36 and 48 h germ groups (24HAIG, 36HAIG and 48HIAG) and the radicle group (24HAIR, 36HAIR and 48HAIR). Finally, all the above materials were stored in liquid nitrogen for subsequent RNA extraction. The germination test of maize inbred line B73, rice cv. “Nipponbare”, switchgrass cv. “Alamo” and orchardgrass cv. “Dianbei” seeds was carried out under the same culture conditions and the same naming rules as above were used. The germinated seeds were counted every 24 h. The germination time of 50% of the seeds in the petri dish and the germination time of all the remaining seeds were recorded.

### RNA extraction and transcriptome sequencing

Total RNA was extracted using the Direct-zol™ RNA MiniPrep Kit (Zymo Research Co.), following the manufacturer’s instructions. After that, RNA purity, concentration and integrity were detected via the NanoPhotometer^®^ spectrophotometer (IMPLEN, CA, USA), Qubit^®^ RNA Assay Kit in Qubit^®^ 2.0 Fluorometer (Life Technologies, CA, USA) and Nano 6000 Assay Kit and Agilent Bioanalyzer 2100 system (Agilent Technologies, CA, USA).

A total of 3 μg RNA from each sample as a sequencing input material, and the detailed method refers to our previous research [70, 71]. First strand cDNA synthesis was implemented by using random hexamer primer and M-MuLV Reverse Transcriptase (RNase H-), and second strand cDNA synthesis was implemented by using mixture containing DNA Polymerase I and RNase H. Total 24 sequencing libraries were generated using NEBNext^®^ Ultra™ RNA Library Prep Kit for Illumina^®^ (NEB, USA) [72]. In order to ensure the quality of bio-analysis, raw reads containing adapters and low quality must be filtered to obtain clean reads for subsequent analysis. The filter criteria are as follows: (1) remove reads with adapters. (2) Remove *N* (*N* means that the base type cannot be determined) is greater than 10% of reads. (3) Remove low-quality reads (reads with *Q*_phred_ ≤ 20 bases accounting for more than 50% of the entire read length). At the same time, the GC content of clean reads, and the values of *Q*20 and *Q*30 were also calculated.

### Quantification of gene expression level and identification of DEGs

We use the Pacbio full-length transcriptome data of pearl millet as a reference sequence for analysis [8]. The classic transcriptome data processing includes two steps: sequence alignment and transcription abundance calculation. This process uses two software, TopHat2 and Cufflinks, but the combination of these two software needs high requirements on computer hardware and is very time-consuming. Here we used kallisto software for a new RNA sequence quantization method, which is close to the best in speed and accuracy [73, 74]. The analysis method refers to the description in the article, briefly, using the cDNA data of the reference sequence to construct an index, and then to identify and quantify the transcript. The expression abundance of genes is expressed by transcripts per million (TPM). The calculation formula of this method is: TPM_*i*_ = (*N*_*i*_/*L*_*i*_) * 1,000,000/sum (*N*_*i*_/*L*_*i*_+……+*N*_*m*_/*L*_*m*_). *N*_*i*_: the number of reads mapped to gene *i*; *L*_*i*_: the total length of the exons of gene *i*. By ensured the same total TPM in each sample, this method can reflect the true expression level of genes more accurately [75]. In order to ensure the accuracy and reliability of the experiment, we filtered out the genes with the maximum TPM less than 5 in the 24 samples. The differential gene identification software used with kallisto software is an R package called sleuth, which can quickly and accurately calculate the differentially expressed genes [76]. Genes with log2 (Group1/Group2) ≥ 1 and the *Q* value < 0.05 obtained by Sleuth software are considered to be differentially expressed.

### WGCNA analysis and gene function annotation

Due to the large number of sampling points in this study, we used R software to perform WGCNA [77]. 24,307 genes were selected for WGCNA analysis. They were screened out by two conditions: (1) In all samples, there was a TPM value greater than 5; (2) The absolute value of log2 (Group1/Group2) ≥ 1 in at least one comparison group. The three key parameters are set as follows: (1) Soft threshold power is 17; (2) MinModuleSize is 30; (3) MergeCutHeight is 0.15. For functional annotations of genes, refer to the annotations in the pearl millet Pacbio full-length transcriptome [8]. For the genes in the modules, we use KOBAS software for KEGG enrichment analysis [78].

### GA content determination

We used the same sampling method and strategy like RNA-seq to obtain samples to measure the GA content, the difference is that two time points 4 and 6 h after seed imbibition were added. Using the same naming rules, the samples at the two time points were named 4HAIS and 6HIAS. We used enzyme-linked immunosorbent assay to determine the content of GA [79]. The Plant Gibberellic Acid (GA) ELISA Kit is provided by Shanghai Enzyme-Linked Biology Company. The experimental method was carried out strictly in accordance with the instructions. Three biological replicates and three technical replicates were measured for each sample.

## Supplementary Information

**Additional file 1: Figure S1.** Morphological changes of four other plants seed from dry seed to seedling. **Figure S2.** Correlation between different samples. **Figure S3.** Pathways shared among the four modules. **Figure S4.** Heat map of genes expression related to brassinosteroid biosynthesis.

**Additional file 2: Table S1.** Germination time of different plant seed. **Table S2.** Length of germ and radicle in different time points. **Table S3.** Summary of raw sequencing data quality. **Table S4.** Correlation coefficient between biological replicates of different samples. **Table S5.** Pathways of gene enrichment in the four modules. **Table S6.** Gibberellin content at different time points. **Table S7.** The original reads counts value of the genes.

## Data Availability

The raw sequence data have been deposited in the NCBI database: PRJNA670183 (https://www.ncbi.nlm.nih.gov/sra/PRJNA670183).

## Abbreviations

KEGG: Kyoto Encyclopedia of Genes and Genomes
ABA: Abscisic acid
GA: Gibberellin
CTK: Cytokinin
DLI: Daily light integral
DEGs: Differently expressed genes
WGCNA: Weighted gene co-expression network analysis
NCBI: National Center for Biotechnology Information
IAA: Indole acetic acid
LHC: Light-harvesting complex
BRs: Brassinosteroids
cDNA: Complementary DNA
RNA-Seq: RNA sequencing
TPM: Transcripts per million
