# Supplementary material for: Transcriptome analysis revealed the regulation of gibberellin and the establishment of photosynthetic system promote rapid seed germination and early growth of seedling in pearl millet
Source: Biotechnol Biofuels. 2021 Apr 11;14:94. doi: 10.1186/s13068-021-01946-6 (PMC8040237; doi:10.1186/s13068-021-01946-6)
Supplement: Supplementary file 1 — Additional file 1: Figure S1. Morphological changes of four other plants seed from dry seed to seedling. Figure S2. Correlation between different samples. Figure S3. Pathways shared among the four modules. Figure S4. Heat map of genes expression related to brassinosteroid biosynthesis. [file 13068_2021_1946_MOESM1_ESM.docx]

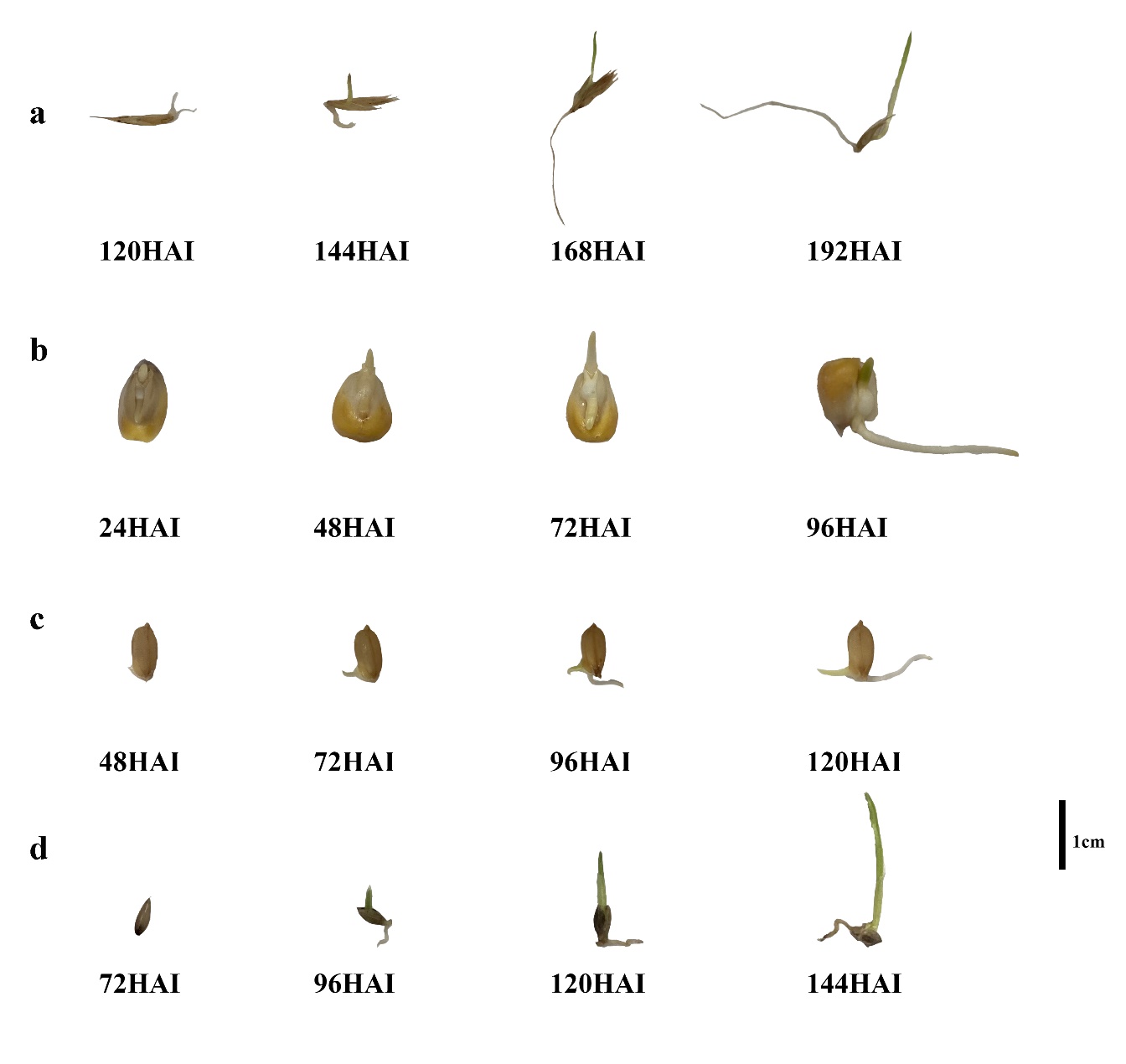


Figure S1 Morphological changes of four other plants seeds from dry seed to seedling. a. Orchardgrass b. Maize c. Rice d. Switchgrass. Note: Due to the different germination time of the four kinds of plants and limited space, we only provide photos from the emergence of the germ to the next three days


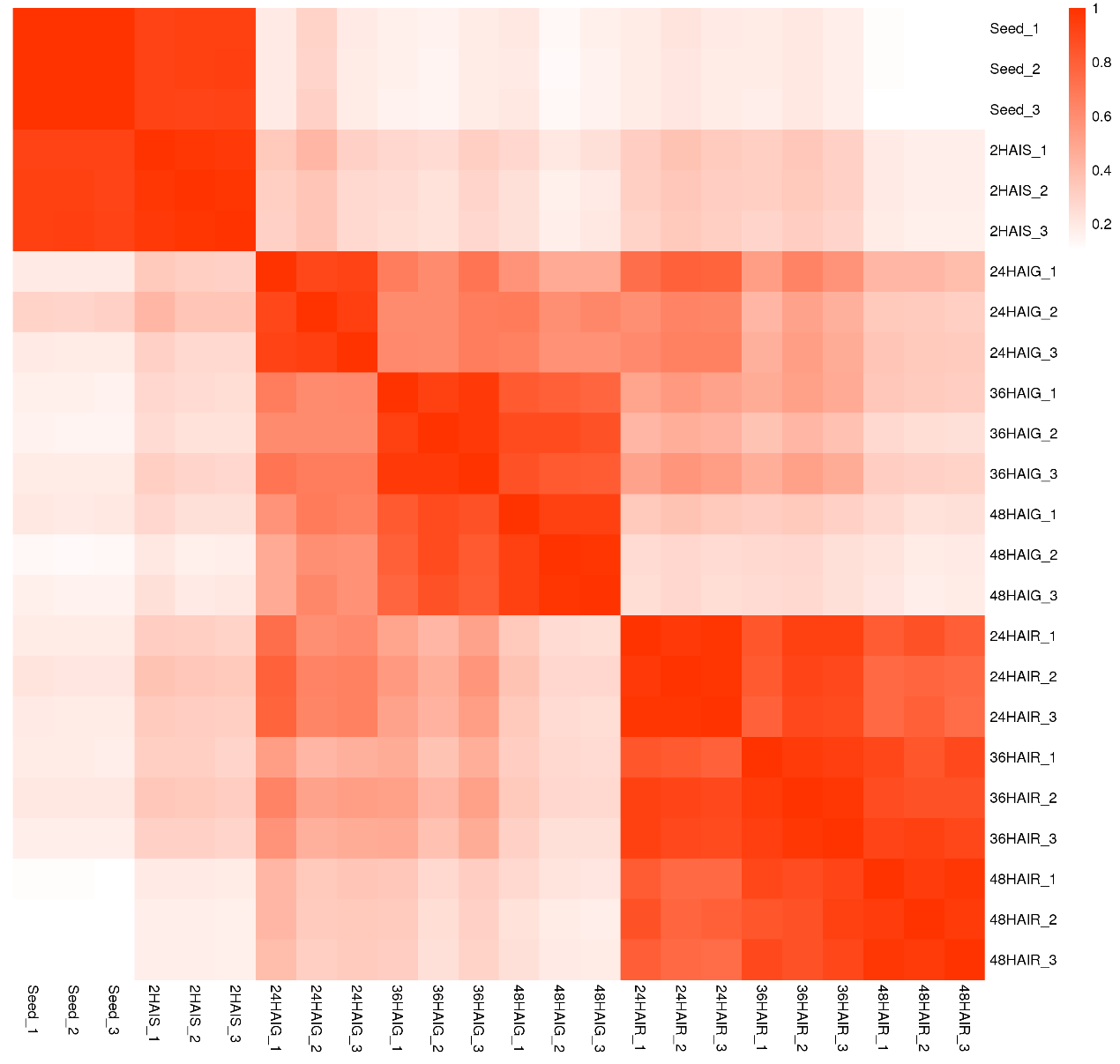


Figure S2 Correlation between different samples


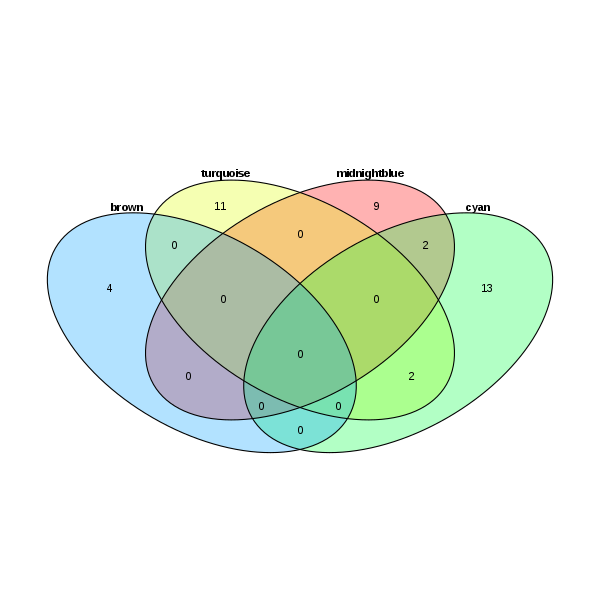


Figure S3 Number of pathways shared among the four modules “brown”, “turquoise”, “midnightblue” and “cyan”

**
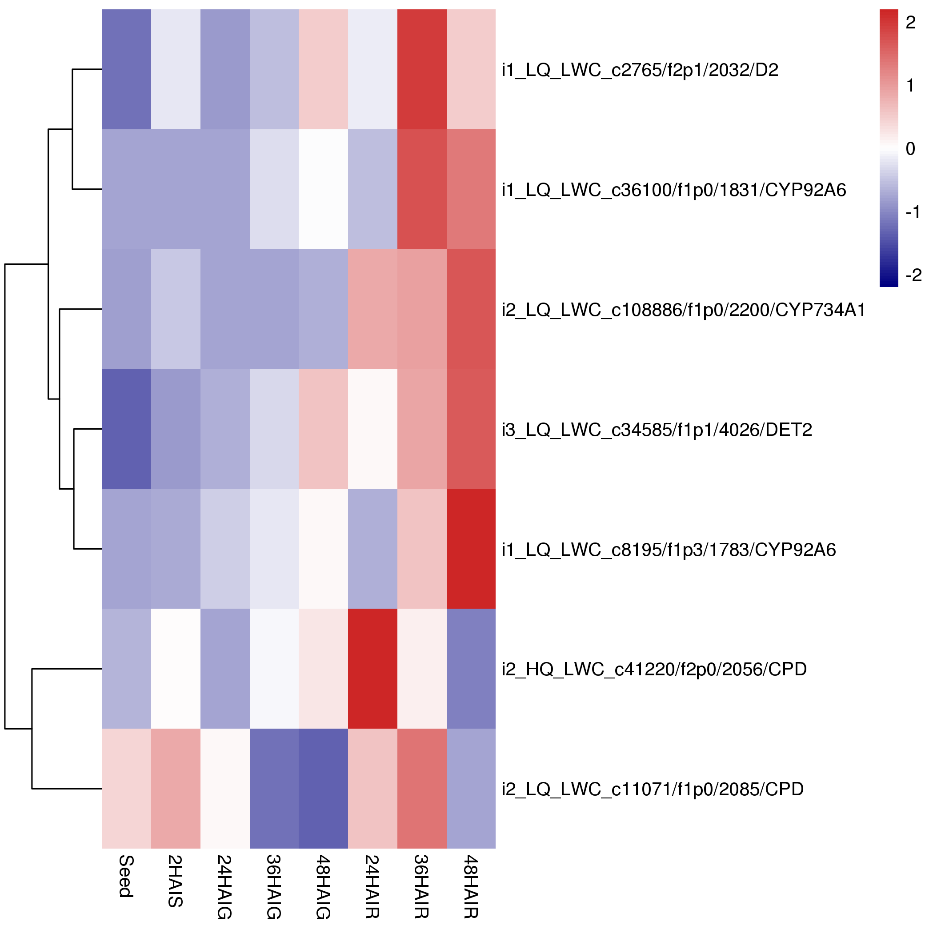
**

Figure S4 Heat map of genes expression related to brassinosteroid biosynthesis. The expression data are the TPM values of the samples, red color indicates upregulated expression, and blue indicates downregulated expression
